# Protocol of the PROMOTE study: characterization of the microbiome, the immune response, and one-carbon metabolism in preconceptional and pregnant women with and without obesity (an observational subcohort of the Rotterdam Periconception cohort)

**DOI:** 10.1371/journal.pone.0319618

**Published:** 2025-04-02

**Authors:** Nicole Schenkelaars, Lieske Wekema, Marijke M. Faas, Régine P.M. Steegers-Theunissen, Sam Schoenmakers

**Affiliations:** 1 Department of Obstetrics and Gynaecology, Erasmus Medical Center, Rotterdam, The Netherlands; 2 Department of Pathology and Medical Biology, University Medical Center Groningen and University of Groningen, Groningen, The Netherlands; UNAM FACMED: Universidad Nacional Autonoma de Mexico Facultad de Medicina, MEXICO

## Abstract

**Introduction:**

Preconceptional and maternal obesity are well-known risk factors for pregnancy and fetal complications including gestational diabetes, hypertensive disorders, and macrosomia. Maternal obesity is associated with offspring obesity and increased healthcare costs. To disrupt the cycle of obesity, we aim to investigate the impact of the composition of the maternal microbiota (bacteria and viruses) throughout preconception and pregnancy and the associations with the immune responses and one-carbon metabolism (1-CM) as an underlying mechanism in the pathophysiology of increased adverse pregnancy outcomes in maternal obesity.

**Methods and analysis:**

The PROMOTE study is a subcohort of the Rotterdam Periconceptional Cohort, a hospital-based observational cohort study. We will include 70 women per BMI group: ≥ 30 kg/m^2^ or 18.5-25 kg/m^2^, at different time points in each group: 10 preconceptional, 50 in the first trimester (with longitudinal follow-up during pregnancy, delivery and postpartum) and 10 in the third trimester of pregnancy. Which makes a total of 140 inclusions. Vaginal and rectal bacteriome, virome, and blood samples are collected. In the third trimester inclusions, only faecal samples are collected. Microbiota samples will be analysed using 16S rRNA sequencing. Bacteriome and virome profiles are compared between the BMI subgroups, associations with general immune responses and 1-CM markers will be shown.

**Trial registration:**

ClinicalTrials.gov (NCT05754645)

## Introduction

In Northwestern Europe the prevalence of obesity, defined as a body mass index (BMI) of ≥ 30 kg/m^2^, increased from 8.5% to 19.4% in 2016 [1]. More specifically, in the Netherlands, the percentage of women with obesity of reproductive age has increased from 6.8% in 1990 to 16.1% in 2022 [2]. With approximately 170.000 births per year in the Netherlands, this results in 25.000-30.000 pregnant women with obesity per year [3].

Preconceptional and maternal obesity (MOB) are well-known risk factors for pregnancy complications including gestational diabetes mellitus (GDM) and hypertensive disorders of pregnancy (HDP), as well as fetal complications such as macrosomia [4]. Moreover, MOB and higher pre-pregnancy BMI are associated with offspring obesity later in life and the associated healthcare costs [5,6]. To break the vicious cycle of obesity, we investigate the role of the maternal gut and vaginal microbiome during the preconceptional period, pregnancy, and delivery as underlying mechanism of deranged 1-CM and oxidative stress, leading to vascular dysfunction and increased risks of gestational hypertension, preeclampsia and gestational diabetes in women with obesity.

A healthy gut microbiota consists of more than 1000 phylotypes which play an important role in health and disease [7]. Their composition is highly variable and modulated by numerous factors including diet, lifestyle, toxins, medication, and diseases [8,9]. Moreover, the microbiota itself can influence many physiological processes, including the immune response and in particular the micronutrient-dependent one-carbon metabolism (1-CM) [10]. Literature has shown a correlation between the altered gut microbiota of pregnant mice and the pregnancy-induced adaptations in the immune responses in order to tolerate the semi-allogeneic fetus [11]. The 1-CM is important during the periconceptional period and pregnancy, because it provides the substrates for the biosynthesis of DNA, RNA, lipids, and proteins, necessary for cell replication and differentiation, as well as moieties for epigenetic programming. The gut microbiota and 1-CM are interlinked, as the gut bacteria contribute to the production and availability of B vitamins, essential substrates of the 1-CM. On the other hand, 1-CM-dependent B vitamins support bacterial survival and maintain gut homeostasis. Similar to the gut microbiota composition, the vaginal microbiota are subject to several factors as well, including sexual activity, hygiene, menstruation and hormone levels, but also pregnancy affects its composition [12]. Interactions between the gut and vaginal microbiota are very limitedly described in literature and research is required to further profile the microbial and viral interplay.

Obesity is associated with gut dysbiosis, characterized by an increased *Firmicutes*-to-*Bacteroidetes* ratio, also observed in pregnant women with obesity [13]. A change in microbiota composition towards *Firmicutes* affect the metabolic potential of the microbiome and leads to an increased capacity to harvest energy from the diet. Studies have investigated the causal relationship between obesity and the gut microbiota, by demonstrating that colonization of germ-free mice with an ‘obese gut microbiota’ causes a greater increase in total body fat than colonization with ‘lean microbiota’. Suggesting that the gut microbiota composition contributes to the pathophysiology of obesity [14].

Therefore, we hypothesize that dysbiosis of the gut microbiota in pregnant women with obesity is considered a chronic endogenous stressor, inducing a deranged 1-CM and altered immune response. These biological derangements contribute to oxidative stress, vascular and placental dysfunction, resulting in increased risks for HDP and GDM [15,16]. Additionally, epigenetic placental and fetal changes and vertical transfer of dysbiotic gut and vaginal microbiota can ultimately accumulate and result in macrosomia and childhood obesity [17].

## Materials and methods

### Aims and objectives

We aim to investigate whether dysbiosis of the maternal gut microbiota and the interactions with vaginal microbiota, in pregnant women with obesity is an underlying mechanism in the pathophysiology of adverse maternal and offspring outcomes. Therefore we analyze the changes in the gut and vaginal microbiome (bacteriome and virome), maternal and fetal immune responses, and 1-CM in pregnant women with obesity in comparison to normal-weight pregnant women. The study objectives include:

1. Identification of the composition of the gut and vaginal microbiota in women with and without obesity.
  1.1. Determine changes in the composition from preconception to pregnancy and longitudinally across the trimesters of pregnancy.
  1.2. Identify interactions between the bacteriome composition of the gut and vagina and the gut virome.
  1.3. Identify differences in the bacteriota composition of the meconium in neonates from women with and without obesity.
2. Identification of a relationship between the gut microbiota (bacteria and viruses), the maternal immune response, and 1-CM in pregnant women.
  2.1. Identify differences in markers of the general immune response and 1-CM in women with and without obesity.

### Study design

#### Setting.

The PROMOTE cohort is a single-center subcohort embedded in the Rotterdam Periconception Cohort (Predict study), conducted in the Erasmus MC, University Medical Centre, Rotterdam, The Netherlands [18,19]. The study visits for the PROMOTE are largely combined with the visits for the Predict study and take place preconceptionally, in the consecutive trimesters of pregnancy, during delivery and postpartum. The setting will be mainly at the outpatient clinics of the department of Obstetrics and Gynaecology, partly at the delivery ward in the Erasmus MC Sophia Children’s hospital, and partly at home. Inclusions started on 21^st^ of July 2022 and are currently ongoing. The study schedule is planned for a period of 60 months, all study participants are expected to have completed all study moments by July 2027. The PROMOTE study is part of a collaboration with the Department of Pathology and Medical Biology, University of Groningen and University Medical Center, Groningen, The Netherlands. This collaboration is based on a jointly obtained ZonMw grant of the Open Competition 2018 (09120011910046).

#### Population.

The PROMOTE subcohort is part of the Predict study population, which includes preconceptional or pregnant women (with gestational age (GA) below 10 weeks), between the ages of 18 and 45. The extensive study protocol for the Predict study is available online [18,19]. Participants included in the PROMOTE subcohort additionally meet the following inclusion criteria: a BMI ≥ 30 kg/m^2^ (group 1) or a BMI between 18.5 – 25 kg/m^2^ (group 2), understanding of the Dutch language, and the willingness to provide written informed consent. Of the total number of inclusions, 20 participants will be included in the third trimester (≥28 weeks of GA). Exclusion criteria of the PROMOTE subcohort comprise factors that are known to influence the gut and/or vaginal bacteriome and virome composition and include: African origin, gastro-intestinal diseases, liver, pancreas, and kidney diseases, pre-existent diabetes mellitus, tobacco use, medication affecting the gut and/or vaginal microbiota and antibiotic use less than 2 weeks before the first sampling. Participants or patients were not involved in the design of the study.

#### Recruitment of participants.

**Fig 1:**
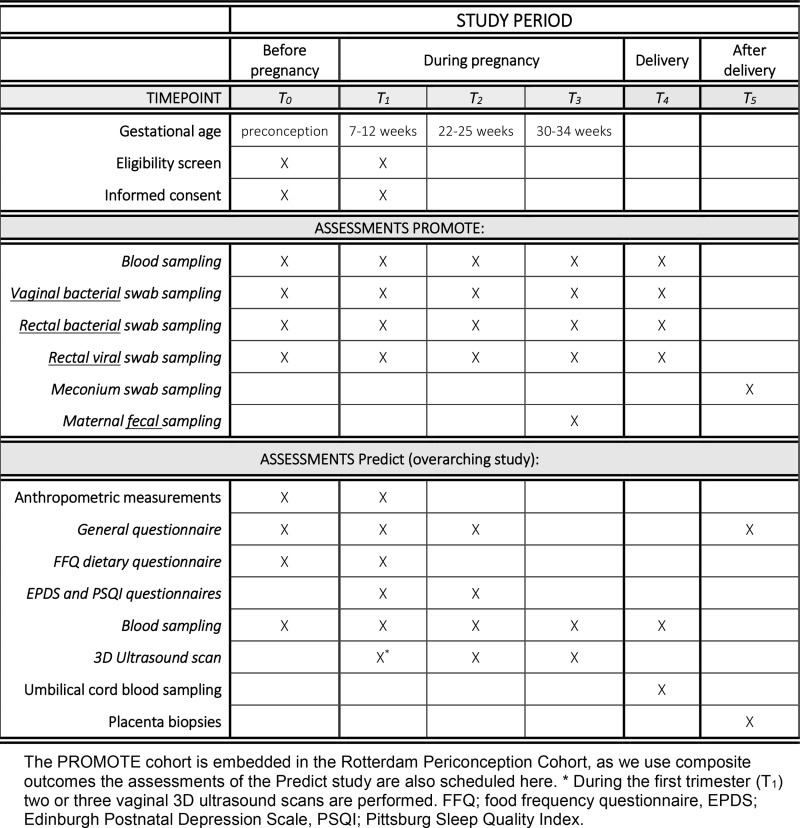
Study Scheme with visits and assessments. Circled in red are assessments conducted as part of the PROMOTE subcohort. GA; gestational age. ^1^ 3D-ultrasound is performed 2-3 times during the first trimester at gestational age 7 weeks, 9 weeks, and 11 weeks. ^2^ Fecal sample collection is only performed in a subcohort of 20 third trimester inclusions of the PROMOTE. ^3^ Rectal and vaginal bacteriome and virome and meconium sample collection is only performed when delivery takes place in the Sophia Children’s Hospital, Erasmus MC..

Participants will be recruited from July 2022 until all inclusions are achieved, expected in mid-2024. The total recruitment target will be 140 women: *n* = 20 preconceptional (subgroup A), *n* = 100 during pregnancy (subgroup B) (Fig 1). Additionally, we will include the neonates of the first trimester inclusions (maximum of *n = *100), when delivery takes place at the Erasmus MC Sophia Children’s Hospital. Furthermore, we will include 20 participants in the third trimester of pregnancy (subgroup C). This makes a total of 140 inclusions of the women and another maximum of 100 inclusions of the corresponding neonates. Two inclusion arms are defined: women with a BMI ≥ 30 kg/m^2^ (group 1) and women with a BMI between 18.5 – 25 kg/m^2^ (group 2). Half of the inclusions will be in the case group (1) and half in the control group (2). A power calculation is not performed, because we conducted this pilot study as an exploratory study to assess the largely unknown variance of the microbiota in pregnant women with obesity. The expected variance of the composition of the gut microbiota, which is highly variable and largely unknown in women with obesity during pregnancy. The few studies that report significant differences in microbiota (on genus and phylum level) of women with and without obesity during pregnancy, include much smaller sample sizes [20–22]. These studies provide strong evidence for the feasibility of our approach and the likeliness to find differences between the groups of BMI.’ Eligible participants will be approached by their healthcare provider/research nurse at Erasmus MC or via the midwife practices in the Rotterdam Rijnmond region and they will receive a study flyer. Women who are willing to participate will be given a patient information folder (PIF) and consent forms (S1 File, translated from Dutch into English). Inclusion and written informed consent will take place at the Erasmus MC, performed by the research nurse.

#### Study procedures.

Women included in the first trimester are followed-up longitudinally during pregnancy, delivery and postpartum which leads to a study duration of approximately 12 months. Women included in the preconception period and third trimester only have one study visit without follow up. Preconceptional included women who conceive pregnancy are allowed to continue the study in pregnancy. During the study period, various measurements and assessments will be conducted. As the PROMOTE subcohort is embedded in the Predict study, assessments conducted as part of the Predict study are also shown in Fig 1. Women included in subgroup A will have one study visit. Women included in Subgroup B will have (a maximum of) 7 study visits: two to three visits in the first trimester (between 7-12 weeks of GA), one visit in both the second (between 22-25 weeks of GA) and third trimester (between 30-34 weeks of GA), one during delivery (if delivery takes place in the Sophia Children’s Hospital, Erasmus MC), and one during postpartum check-up (if check-up takes place in Erasmus MC, ≥ 6 weeks postpartum). Women included in subgroup C will have two study visits (≥30 weeks of GA), which coincide with their regular obstetric care check-ups.

#### Maternal characteristics.

Data on maternal characteristics were extracted from validated questionnaires and include lifestyle, supplement use and dietary patterns, in order to correct for potential confounding factors.

Data was collected on variables that possibly influence the outcomes, using validated patient-reported questionnaires. These variables included among others; maternal age, cigarette and alcohol use, mode of conception categorized in natural conception and in vitro fertilization (IVF)/intracytoplasmic sperm injection (ICSI), country of origin categorized as the Netherlands, Europe, or other according to the Dutch Central Bureau of Statistics (CBS) [23]. Information on the dietary pattern, including the amount of sugar and fat in the participants diet are analyzed from the food frequency questionnaire.

#### Sample collection.

In subgroup B, two rectal (one bacteriome and one virome) and one vaginal swab sample (bacteriome) are collected at each study visit and during delivery. Swabs are self-collected by the participants, according to standardized protocols, see the instruction card (S2 File). During delivery, the caretaker is asked to collect the maternal swabs and a meconium sample. After collection, the samples are immediately snap-frozen and stored at -80˚C. Blood sampling will take place at the outpatient clinic during each study visit or submission at the delivery ward using vacutainer tubes. One 6 mL serum tube, one 6 mL EDTA tube, and two 10 mL heparin tubes are collected. All samples are pseudo-anonymously coded with a corresponding study number in the Erasmus MC, Rotterdam. The final trial dataset is accessible for the research team of the PROMOTE study. An extensive description of the sample collection and assessments conducted as part of the Predict study can be found in the Cohort Profile (update) papers by Steegers-Theunissen and Rousian et al. [18,19]. Faecal samples are collected from 20 women in subgroup C. They are asked for study participation during regular care check-ups at the obstetrics outpatient clinic or during admission at the obstetrical ward of the Sophia Children’s Hospital, Erasmus MC, Rotterdam. After informed consent is provided, they receive a feces collection kit from their caretaker in order to collect feces at home or during admission, according to our standardized instructions (S3 File). Fecal samples are frozen in the regular freezer of participants at -20˚C and transported in a Styrofoam box to their next regular appointment. Here they are received by the researcher and stored at -80˚C. Fecal samples from women that are admitted are collected by the researcher and immediately snap-frozen at -80˚C. All collected data will be stored with the use of data management systems, including CASTOR. All data will be encrypted, using a study number. The key between the patient identification number and the study number is only available to the researchers directly involved in the study. Regarding handling missing data, participants only having one sample moment (subgroup A or C) will be excluded from the study if the sample does not contain sufficient DNA for analysis. Participants which are having longitudinal follow-up sampling moments (subgroup B) will not be excluded from all analysis, if there is a missed sampling moment, only the missing sample will be left out. The missing data of subgroup B will be systematically reported in the results section. In order to minimize researcher bias during sample analysis, the samples are blinded by a study number, ensuring that researchers are unaware of the BMI category. Regarding potential sampling bias, we standardized collection procedures and utilize well-established methods for microbiome analysis, using bulk sequencing to avoid badge differences. Furthermore, in order to minimize researcher bias during sample analysis, the samples are blinded by a study number, ensuring that researchers are unaware of the BMI category.

### Laboratory assessments

#### Blood samples.

Serum and EDTA tubes will directly after collection be centrifuged and separated into serum and plasma aliquots by the Trial laboratory of the Erasmus MC, according to standardized lab protocols. Afterwards, they will be stored at -80˚C at the Central Cold Storage in the Erasmus MC. EDTA tubes are transported on ice, to ensure valid assays of homocysteine. Determination assays will be performed at the moment all samples have been collected from the total study population. Laboratory assessments will include metabolic indicators (glucose, insulin, cholesterol, HDL, LDL, lipids) using standard methods. Cytokines (TNF-α and IL-6) using Luminex, hsCRP using ELISA, and parameters of 1-CM (folate, homocysteine, and vitamin B12). To avoid ceiling effects in immune and metabolic assays, we will first perform a dilution series with a subset of samples to identify the linear range of the assay. All samples will be measured in serial dilutions and in duplo. To avoid flooring effects, we will use high sensitive assay if necessary. Blood in heparin tubes is collected by the researcher immediately after withdrawal, from which Peripheral Blood Mononuclear Cells (PBMCs) are isolated, according the followed protocol (S4 File). Isolated PBMC cells are frozen at -80˚C using the Thermo Scientific ^TM^ Mr. Frosty ^TM^ Freezing Container. Within 24 hours after freezing the PBMCs are transferred to the liquid nitrogen freezer.

#### Microbiome samples.

To avoid badge differences, DNA from fecal samples will be isolated and sequenced in bulk whenever possible. For DNA isolation of rectal and vaginal swab samples, 1 ml of the buffer is used, for fecal samples, 0.25 gram feces is used. The extensive protocol used for DNA isolation are available via the S5 File. The bacteriome profiles will be assessed by 16SrRNA gene amplicon sequencing by the company Novogene. Bacterial 16S rRNA gene sequences covering variable regions (V3-V4) of the 16S rRNA gene will be amplified using specific barcoded primers. Before sequencing, quality of the samples will be checked for degradation; only samples with no degradation will be used. After library preparation, short-read sequencing will be done using the Illumina platform. Sequences will be analysed using the Amplicon Sequence Variant (ASV). QIIME2’s classify-sklearn algorithm will be applied for species annotation of each ASV [24,25]. The annotation database that will be used is Silva 138.1 [26,27]. Virome samples will be analyzed according to standardized protocols by the Department of Viroscience Erasmus MC (S6 File). For metagenomic sequencing, 110 μL of sample material will be centrifuged at 10.000 xG for 10 min and 100 μL of the supernatant will be treated with 20 U of Turbo DNase (Invitrogen) for 30 min at 37°C. Nucleic acids will be isolated using the High Pure Viral Nucleic Acid Kit (Roche). cDNA will be made using SuperScript IV (Invitrogen) and it will be made double stranded with Klenow Fragment (3’à5’ exo, 5U/μL) (New England Biolabs). Sequencing libraries will be generated using the Kapa HyperPlus Kit (Roche): First the samples are fragmented, followed by an end repair and A-tailing. Then dual-indexed adapters are being attached. After a bead clean-up and a double-sided size selection with AMPure XP beads (Beckman Coulter) a pre-capture LM-PCR will be performed. The sample library is then purified and the concentration is measured with a Qubit DS DNA High Sense assay (Invitrogen) and the sample size is measured on a Tape Station (Agilent). A maximum of 96 metagenomic samples can be pooled. The samples are then being sequenced on a NovaSeq flow cell (Illumina). With respect to consistency and reproducibility across different labs: all microbiome measurements, immune and metabolic assays will be done at the same lab and sequenced or measured in bulk whenever possible.

#### Faecal samples.

Frozen faecal samples are only collected as part of this study, the processing and analysis are performed at the Department of Pathology and Medical Biology, University of Groningen and University Medical Center, Groningen. This stems from the fact that the study is part of a collaboration with the University Medical Center Groningen (UMCG) based on a jointly obtained Open Competition ZonMw grant (09120011910046). The fecal samples are transported to the UMCG and analyzed according to standardized ethically approved study protocols by the Central Committee on Animal Testing. The protocols are available upon request.

### Outcome measures

The main outcome measures are the compositions (alpha- and beta-diversity) of the maternal gut/vaginal bacteriome and virome in women with obesity and women without obesity during the preconception, pregnancy, antepartum and postpartum period. Secondary study outcomes include associations with maternal immune response and 1-CM (e.g., inflammatory and biomarkers) and the composition of the bacteriome/virome. Furthermore we will show clinical maternal characteristics (e.g., GA at delivery, hypertensive disorders of pregnancy), maternal conditions and lifestyle (e.g., diet, medication use, working activities, physical activity, intoxications), clinical fetal outcomes (e.g., growth trajectories in the first, second and third trimester, birthweight), and placental function (e.g., placental weight, inflammatory status).

### Statistical analysis

Descriptive statistics for maternal and lifestyle conditions will be presented to provide a general overview of the data. These conditions include but are not limited to medication use, intoxications, infections, physical activity, working activities, blood pressure, nutrition, and folic acid supplement use. Mean and standard deviation are used for variables that are approximately normally distributed, median and interquartile range for variables that do not appear to have a normal distribution. Categorical variables will be presented as counts and proportions. The independent samples t-test and Mann-Whitney U test will be used for continuous data.

#### Microbiome.

The microbiota will be investigated using different analyses. The alpha diversity (microbiota species within a sample) will be described using the Shannon-index. We will use univariate analysis to describe and compare alpha-diversity per person and per analysis group (1 and 2) by chi-square test. These descriptions will be presented and compared at different time points (preconception, first trimester, second trimester, third trimester, antepartum and postpartum). The Shannon index will be calculated for the analysis of alpha (within-sample) diversity on ASV level. Differences in relative abundances, Shannon indices and *Firmicutes-*to-*Bacteroidota* ratios between the BMI categories will be tested using the Mann-Whitney U test followed by the Benjamini-Hochberg procure in order to calculate the False Discovery Rate (FDR) for conducting multiple comparisons. The alpha diversity will further be analyzed using a marginal model for repeated measurements (fitted using generalized least squares). BMI-group, time, and their interaction will be included as covariates in this model as well as the presumed confounders (including but not limited to: parity, maternal age, mode of conception, fetal gender, diet and lifestyle). Several common variance-covariance structures will be used and the one that achieves the best fit according to the Akaike information criterion will be used for further analyses. A likelihood ratio test will be used to test the hypothesis that the evolution of the alpha-diversity differs between the BMI groups. Beta-diversity (variation of microbial communities between the samples) will be presented using Principal component analysis. We will also analyze differences at the phylum and genera level between time units (preconception, first trimester, second trimester, third trimester, antepartum, postpartum) per group, between the 2 groups of persons (1 and 2) at the different time units (first, second and third trimester, antepartum and postpartum). Differences between groups will be tested using permutational multivariate analysis of variance (PERMANOVA). P-values will be considered significantly different if < 0.05.

#### Secondary study outcomes.

Exploratory analysis will be used to identify associations between the gut microbiota and immune cell changes during pregnancy in obese women and women with normal weight. Individual microbiota abundances of the third trimester with immune cell data, 1-CM markers and other continuous outcomes (birth weight, placental weight) of the same woman will be correlated using the spearman’s correlation coefficient. The correlation coefficients will be visualized in clustered heat maps using Euclidian distance and Ward’s clustering method. A p-value < 0.05 will be considered statistically significant unless otherwise stated. Models will be adjusted for potential confounders (including, but not limited to: parity, maternal age, mode of conception, fetal gender, diet and lifestyle). Corrections for multiple testing are made, to minimize the risk of type 1 error. The interpretation of these exploratory results will not be presented as definitive evidence, but as pointers to future hypotheses to be tested in more rigorous, confirmatory studies.

#### Ethical considerations.

This study was registered prospectively (NCT05754645) after ethical approval was received for version 2.3 in July 2022 and amendment A0001-A0003 by the Medical Ethics Committee (METC) Erasmus MC, Rotterdam, The Netherlands. For the World Health Organization Trial Registration Data Set see Table 1. Any study amendments will be submitted for approval by the METC. The study will be conducted according to the principles of the Declaration of Helsinki (version October 2013) and in accordance with relevant national guidelines, regulations, and Acts (e.g., for Erasmus MC the Medical Research Involving Human Subjects Act (WMO). A monitoring plan has been established with an independent monitor of the data monitoring committee of the Erasmus MC, and will be conducted if 70% of the inclusions are achieved.

**Table 1:** World Health Organization Trial Registration Data Set.

Data category	Information^32^
Primary registry and trial identifying number	ClinicalTrials.gov NCT05754645
Date of registration in primary registry	3 June, 2023
Secondary identifying numbers	NL80155.078.22/OZBS72.21318
Source(s) of monetary or material support	ZonMw grant Open Competition 2018 (09120011910046) The Department of Obstetrics and Gynecology, Erasmus MC, University Medical Center, Rotterdam, The Netherlands
Primary sponsor	ZonMw grant Open Competition 2018 (09120011910046)
Secondary sponsor(s)	The Department of Obstetrics and Gynecology, Erasmus MC, University Medical Center, Rotterdam, The Netherlands
Contact for public queries	Dr. Sam Schoenmakers, MD, PhD [s.schoenmakers@erasmusmc.nl]
Contact for scientific queries	Dr. Sam Schoenmakers, MD, PhD The Department of Obstetrics and Gynecology, Erasmus MC, University Medical Center, Rotterdam, The Netherlands
Public title	The Microbiome in (Non-) Obese Pregnancy and Pregnancy Outcomes (PROMOTE)
Scientific title	The PROMOTE study: characterization of the microbiome, the immune response, and one-carbon metabolism in preconceptional and pregnant women with and without obesity (an observational subcohort of the Rotterdam Periconception cohort)
Countries of recruitment	The Netherlands
Health condition(s) or problem(s) studied	Preconceptional maternal obesity and obesity during pregnancy
Intervention(s)	Vaginal and rectal microbiota swabs (viruses and bacteria), blood withdrawal
Key inclusion and exclusion criteria	Ages eligible for study: ≥ 18 years Sexes eligible for study: women Accepts healthy volunteers: no
	Inclusion criteria: adult patient ( ≥ 18 years), contemplating pregnancy or < 13 weeks pregnant, BMI > 30 kg/m^2^ or BMI 18.5-25 kg/m^2^, understanding of Dutch language.
	Exclusion criteria: African origin, Multiple pregnancy, active tobacco use, gastro-intestinal diseases, heart diseases, liver, pancreas or kidney diseases, use of antibiotics < 2 weeks before participation and pre-existent diabetes mellitus.
Study type	Observational Cohort study
	Primary purpose: prevention
Date of first enrolment	July 2022
Target sample size	140
Recruitment status	Recruiting
Primary outcome(s)	Composition of gut and vaginal bacteriota (genera level)
Key secondary outcomes	Composition of gut virota Maternal immune response (inflammatory markers; AOPP, IL-6, TNF-alpha)
	1-Carbon metabolism response (markers; Homocysteine, Vitamin B2, B6, B12.
	Clinical maternal outcomes: hypertensive disorders of Pregnancy, gestational diabetes, placental weight

## Discussion

### Research implications

Increased understanding of the role of the microbiome in health and disease will contribute to an improvement in understanding the (patho-) physiology of pregnancy complications linked to MOB. This study will contribute to elucidate the associations between MOB, the gut microbiota, general immune response, and 1-CM as an underlying mechanism in the risks of maternal and offspring health. With our data, we will first show longitudinal information on gut and vaginal microbiome composition in women with and without obesity. Second, we provide more insight in the interactions in composition of the bacteria and viruses. Third, we aim to elucidate the MOB-related dysbiosis, and the associations with deranged immune responses, as well as 1-CM. Awareness of the role of the microbiome in pregnancy in Obstetrics and Gynaecology may result in new treatment strategies, not only for pregnant women with obesity, but also for pregnancy complications in normal-weight women. Enhanced understanding of the mechanisms underlying biologically deranged immune responses and 1-CM will help to provide insight into how these chronic stressors contribute to vascular and placental dysfunction and ultimately increase the risks of adverse pregnancy outcomes including HDP and GDM, as well as offspring macrosomia and obesity. If differences are found between the composition of gut and vaginal bacteriome and virome, we would like to validate our study on a large scale, where we can also take into account adverse maternal and fetal outcomes.

Healthcare costs for mothers with obesity are 37% higher compared to women with a normal weight, which is a result of complications related to MOB [6]. Additionally, children born to obese mothers visit a physician 10% more frequently, are 16% more often admitted to a hospital, and spend 10% more days during their admission in their first 18 years of life [28]. All of which increase the cost of healthcare for infants by 72% [29]. The results of our project may provide information about the pathophysiology underlying offspring and maternal health and obesity-related healthcare costs, both short and long-term. Simultaneously, we provide the opportunity to develop new treatment strategies with pre- or probiotics, to restore maternal dysbiosis in women with obesity and thereby break the vicious cycle of obesity inheritance and lower the costs of healthcare. As a result, by improving the health of periconceptional women with obesity, maternal, fetal, placental, and neonatal health can all be improved throughout the life course.

### Strengths of this study

- Longitudinal collection of microbiota and virota samples during pregnancy.
- Paired sampling of bacteriota of gut and vagina.
- Sample size of 140 participants.

## Supporting information

S1 File Subject information and informed consent forms. (PDF)

S2 File Manual collection of microbiome swab PROMOTE study. (PDF)

S3 File Manual for collection stool sample PROMOTE study. (PDF)

S4 File Protocol PBMC isolation. (PDF)

S5 File Faecal DNA extraction protocol. (PDF)

S6 File Illumina capture sequencing protocol. (PDF)
