# Supplementary material for: Protocol of the PROMOTE study: characterization of the microbiome, the immune response, and one-carbon metabolism in preconceptional and pregnant women with and without obesity (an observational subcohort of the Rotterdam Periconception cohort)
Source: PLoS One. 2025 Apr 2;20(4):e0319618. doi: 10.1371/journal.pone.0319618 (PMC11964453; doi:10.1371/journal.pone.0319618)
Supplement: S1 File — (PDF) [file pone.0319618.s001.pdf]

S1 File. Subject information and informed consent forms.

NOTE; this file is translated from Dutch to English

Subject information for participation in medical scientific research

PROMOTE study

Official title: *"The PROMOTE study: the characterization of the microbiome in pregnancy and prediction of pregnancy outcomes."*

Introduction

Dear madam, parents-to-be,

With this information letter, we would like to ask you if you (and your child) would like/want to participate in medical scientific research. Participation is voluntary. You are receiving this letter because you are pregnant or contemplating pregnancy and are participating in the Predict study. It tells you what the study is about, what it involves, and what the advantages and disadvantages are. It is a lot of information. Would you like to read through the information and decide if you (and your child) want/want to participate? If participating in pregnancy, both parents are asked to fill out Form B2 from Appendix B.

Ask your questions

You can make your decision with the information found in this information letter. In addition, we recommend the following:

- Ask questions of the researcher giving you this information.
- Talk to your partner, family or friends about this study.
- Ask questions of the independent expert, Dr. Vermeulen, pediatrician/neonatologist.
- Read the information at www.rijksoverheid.nl/mensenonderzoek.

1. General information

This study was designed and is being conducted by the Department of Obstetrics and Gynecology at Erasmus MC in Rotterdam. Researchers, these may be doctors and/or research nurses, conduct the research at Erasmus MC. ZonMw pays for this research. The medical ethics review committee at Erasmus MC has approved this research. This study requires 140 subjects.

2. What is the purpose of the study?

In this study we look at the changes in the vaginal and gut microbiome (such as bacteria, viruses and fungi) during/before pregnancy. In addition, we want to see if the composition of the microbiome is related to the effect on inflammation in the body, for this we are also investigating a subgroup of pregnant women with a BMI >30 kg/m2. This will allow us to predict possible unwanted pregnancy outcomes early in pregnancy and thereby possibly prevent them.

3. What is the background of the study?

There is evidence that an increased level of inflammation can develop in the body of women where the microbiome is (unnoticed) out of balance. This may cause a greater chance of developing poor pregnancy outcomes such as high blood pressure, diabetes and preeclampsia. Too little research has been done on this. If our research shows that a disturbed microbiome is prevalent and carries more risks, we would like to explore future interventions (e.g., pro-biotics) to reduce these risks.

4. How does the study proceed?

This study is part of the Predict and lasts until the end of the pregnancy. After pregnancy, we will ask you to complete one more questionnaire one year after the delivery date. If participating in the Predict before pregnancy, you can also participate in the PROMOTE and the additional measurements will be done once as described below.

Visits and measurements

For the PROMOTE study, during your visits for the Predict study, we will do additional blood draws and microbiome collections. The microbiome collections are vaginal and rectal cultures: these may be taken by you if you wish. This means that in addition to the Predict study:

- Withdrawal of 20-40ml blood during the 1st,2nd and 3rd trimester of pregnancy.
- Take vaginal and rectal swabs during the 1st, 2nd and 3rd trimester (by yourself if desired).
- (If possible) during delivery take blood, swabs of the placenta, vagina and stool and a culture of the baby's first stool (meconium).
- 8 weeks after delivery take blood and vaginal and rectal swabs.

5. What do we expect from you?

When participating in the study, we ask that you keep the appointments made and follow the instructions to the best of your ability. This is very important for the accuracy of the study.

There are no things you need to leave out of this study, however, it is necessary that you contact the researcher if:

- You are hospitalized or treated.
- You no longer wish to participate in the study.
- Your phone number, address or email address changes.
- There are changes in your medication.

6. What are the disadvantages of participating?

The blood draws may be painful or cause bruising: this does not happen often. In addition, taking cultures of the vagina and rectum may feel like discomfort, but this is not harmful.

7. What are the advantages of participating?

Participating in research can have advantages and disadvantages. Below we list them. You do not personally benefit from participating in this study. Your participation contributes to more knowledge about the microbiome in pregnant women and its possible influence on pregnancy and pregnancy outcomes.
Disadvantages of the study may include:

- Possible chance findings about your or your child's health.
- Possible discomfort from the measurements/cultures in the study.
- That you will spend extra time on visits at Erasmus MC.

8. When does the study stop?

The study stops for you when

- All visits have been completed, as described above.
- You want to stop the study yourself. You may do so at any time. Please report this immediately to the researcher. You do not have to say why you are stopping.
- The researcher thinks it would be better for you to stop.
- Erasmus MC, the government or the reviewing medical ethics committee decides to stop the research.

What happens when you decide to quit the study?

The researchers use the data and body material (blood, cultures) collected up to the time of stopping. If you wish, collected body material can be destroyed. Please communicate this to the researcher. The entire study ends when all participants are finished.

9. What happens after the study?

The researcher may let you know the main outcomes sometime after the study is completed. If you do not wish this, please indicate this to the researcher.

10. What do we do with the collected material of you and your child?

Are you participating in the study? Then you consent to the collection, use and storage of your and your child's data and bodily material.

What data do we store?
This includes data such as your (and your child's) name, address, date of birth, and data we collect during the study.

What body material do we store?
We store blood samples, cultures, biopsies of the placenta and umbilical cord.

Why do we collect, use and store data and body material?
We collect, use and store your data and body material to answer the questions of this study. And to publish the results.

How do we protect your (and your child's) privacy?
To protect your privacy, we code your (and your child's) data and body material. We put only this code on all the data and body material. We keep the key to the code in a secure place at Erasmus MC. When we process the data and body material, we always use only this code. Even in reports and publications about the research, no one can recall that it was about you.

Who can see the data?
Some people can see your name and other personal information without a code. These are people who verify that the researchers are conducting the study properly and reliably. These persons can access your data:

- A controller working for Erasmus MC or appointed by Erasmus MC.
- National supervisory authorities. For example, the Health Care and Youth Inspectorate.

These persons will keep the data confidential. We ask you to give permission for this access.

How long do we keep data and body material?
We keep the body material from you (and your child) at Erasmus MC. It will be kept for a maximum of 15 years in order to be able to make new determinations related to this study.

May we use the data and body material for other research?
The data and (remaining) body material of you (and your child) collected as part of this study may also be important for other scientific research in the field of the microbiome and pregnancy after this study is completed. For this purpose, the data and bodily material will be stored at Erasmus MC for 15 years after the end of the study. In the consent form you indicate whether you approve of this. Do you not give your consent? Then you can still participate in this study.

What happens with unexpected discoveries?
During the study, we may happen to find something important to your health. The investigator will then contact the relevant healthcare provider. You will then discuss with your family

doctor or specialist what should be done. With the form, you consent to inform your family doctor or specialist.

Can you withdraw consent to the use of your data?

You (and/or father) can withdraw consent to the use of (you and your child's) data at any time. But please note: If you withdraw your consent, and researchers have already collected data for a study? Then they may still use this data. For bodily material, the researchers destroy this after you withdraw your consent. But have measurements already been taken with body material? Then the researcher may continue to use the results.

Do you want to know more about your privacy?

- Want to know more about your rights when processing personal data? Then visit www.autoriteitpersoonsgegevens.nl.
- Do you have questions about your rights? Or do you have a complaint about the processing of your personal data? If so, please contact the person responsible for processing your personal data at Erasmus MC. See Appendix A for contact information.
- If you have complaints about the processing of your personal data, we recommend that you first discuss them with the research team. You can also go to Erasmus MC's Data Protection Officer or file a complaint with the Personal Data Authority.

Where can you find more information about the study?

You can find more information at the following website: https://www.clinicaltrials.gov/. After the study, this website may show a summary of the results of this study.
You can find the study by searching for: NCT05754645.

11. Will you receive compensation if you participate in the study?

The additional tests for the study cost you nothing. You will receive pregnancy images from the ultrasounds taken in the Predict study as a thank you for your participation.

12. Are you insured during the study?

You are not additionally insured for this study. Because participating in the research has no additional risks. Therefore, Erasmus MC is not required by the Medical Ethical Review Committee (METC) to take out additional insurance.

13. Inform primary care physician, obstetrician and/or attending specialist?

In principle, your treating physician, midwife and family doctor will not receive any results of the study. However, the investigator will discuss any findings that may be of personal relevance to you with you and your treating (family) physician or midwife. If you do not wish your treating (family) physician and/or midwife to be informed of any findings, you may not participate in the study.

14. Questions?

Please direct questions about the study to the research team. Would you like advice from someone who has no interest? Then see the independent doctor. She knows a lot about the study, but does not participate in it. Do you have a complaint? If so, discuss it with the researcher or the doctor treating you. Would you rather not? Then go to your hospital's complaint committee. Appendix A tells you where to find them.

15. How do you give permission for the study?

You can think calmly about this study first. If you decide that you would like to participate then you will complete both consent forms. By doing so, you consent to the collection and use of your own data (Appendix B1) and, if participating during pregnancy, to the collection and use of your child's data (Appendix B2). The use of your child's data requires the consent of both parents; for this reason, we ask that your child's father also give consent by signing Appendix B2. You and the researcher will both receive a signed version of this consent form.

16. Appendixes

A. Contact information for Erasmus MC.
B. Consent forms

Thank you very much for your time and attention!

Kind regards,
The research team of the PROMOTE study

Appendix A: Contact details of the Erasmus MC

If you have questions of complaints, please contact:

General email: promote.studie@erasmusmc.nl

Principal Investigator:
Prof. dr. R.P.M. Steegers – Theunissen, non-practicing physician, epidemiologist, professor Periconception epidemiology
Tel: 06 – 81 17 76 05

Fellow researchers:
Mw. drs. N. Schenkelaars, PhD-candidate
Tel: 06 – 81 17 76 05

Dhr. dr. S. Schoenmakers, maternal-fetal specialist
Tel: 010 – 703 74 39

Independent doctor:
Dr. Marijn Vermeulen, pediatrician, neonatologist
Tel: 06 – 33 33 04 55

Erasmus MC Data Protection Officer:
Erasmus MC's Data Protection Officer can be reached through the secretariat of the Legal Affairs Department, tel. 010 - 703 49 86

Erasmus MC Complaints Committee:
If you are not satisfied with the examination or treatment, you can contact Erasmus MC's independent complaints committee.
The complaints committee can be reached at telephone number 010 - 703 31 98.

Appendix B1: Informed consent form – <u>future mother</u>

"The PROMOTE study: the characterization of the microbiome in pregnancy and prediction of pregnancy outcomes".

- I read the information letter. I was also able to ask questions. My questions were answered well enough. I had enough time to decide whether to participate.
- I know that participating is voluntary. I also know that I can decide at any time not to participate in the study after all. Or to stop. I do not have to say why I want to stop.
- I give permission to inform my doctor/specialist about unexpected findings from the study that are important for my health.
- I give the researchers permission to collect and use my[data and/or body material. The researchers do this only to answer the research question of this study.
- I know that to monitor the study, some people may see all of my data. Those people are listed in this information letter. I give these people permission to see my data for this audit.
- I consent to participate in the studies in the PROMOTE study as stated in the information leaflet
- I give permission for my data to be kept and used for other research on the microbiome, as stated in the information letter. The data will be kept for this purpose for 15 years after the end of the study. Yes ☐ No ☐
- I give permission to keep and use (leftover) body material for other research, as stated in the information letter. The material will be kept for this purpose for 15 years after the end of the study. Yes ☐ No ☐
- I give permission to be contacted again for possible follow-up research.
  Yes ☐ No ☐

Name (participant): ………………………………..

Signature (participant): …………………………… Date: __ / __ / __

I certify that I have fully informed this subject about the said study. Will any information become known during the research that may affect the subject's consent? If so, I will let this subject know in time.

Name researcher (or his representative):………………….……………………………….

Signature:……………………… Date: __ / __ / __

Appendix B2: Informed consent form – parents-to-be

"The PROMOTE study: the characterization of the microbiome in pregnancy and prediction of pregnancy outcomes".

- I read the information letter. I was also able to ask questions. My questions were answered well enough. I had enough time to decide if my child will participate.
- I know that participating is voluntary. I also know that I can decide at any time not to have my child participate in the study after all. Or to stop. I do not have to say why I want to stop.
- I give permission to inform my family doctor/specialist about unexpected findings from the study that are important for my child's health.
- I give the researchers permission to collect and use data and/or body material from my child. The researchers will do this only to answer the research question of this study.
- I know that for the purpose of monitoring the study, some people may see my child's data, as stated in the information letter. I give these people permission to see my data for this monitoring.
- I give permission to participate in the examinations of my child in the PROMOTE study as stated in the information leaflet
- I give permission for my child's data to be kept longer and used for future research on the cause and genesis of pregnancy complications. Yes ☐ No ☐
- I give permission to keep (leftover) body material from my child to use for other research, as stated in the information letter. The material will be kept for 15 years for this purpose. Yes ☐ No ☐
- I give permission to be contacted again for follow-up research regarding my child. Yes ☐ No ☐

Name mother-to-be:
Handtekening: Date: __ / __ / __

Name father-tobe:
Handtekening: Date: __ / __ / __

I certify that I have fully informed this subject about the said study. Will any information become known during the research that may affect the subject's consent? If so, I will let this subject know in time.

Name researcher (or his representative):…………………..……………………………….
Signature:……………………… Date: __ / __ / __

Indien van toepassing:

Additional information was given by:
Name:
Position:
Signature Date: __ / __ / __

A complete information letter will be given to the parent along with a signed version of the consent form.
