# Supplementary material for: Protocol of the PROMOTE study: characterization of the microbiome, the immune response, and one-carbon metabolism in preconceptional and pregnant women with and without obesity (an observational subcohort of the Rotterdam Periconception cohort)
Source: PLoS One. 2025 Apr 2;20(4):e0319618. doi: 10.1371/journal.pone.0319618 (PMC11964453; doi:10.1371/journal.pone.0319618)
Supplement: S2 File — (PDF) [file pone.0319618.s002.pdf]

S2 File. Manual collection of microbiome swab PROMOTE study.

1. Make sure you are undressed from undergarments and are able to collect the swab.
2. Collect the swab according to the instruction below.
3. Make sure the tubes do not fall over! The fluid must NOT be lost.
4. Place the swab back in the plastic container with a <u>V</u> for the vaginal swab and <u>R</u> for rectal swab.
5. Return tubes to researcher.

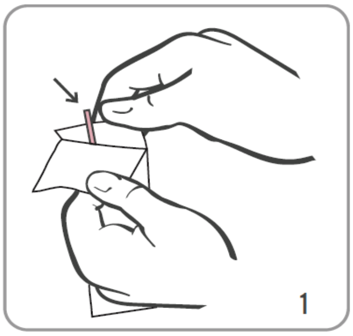

Remove swab from package. Only touch the grip!

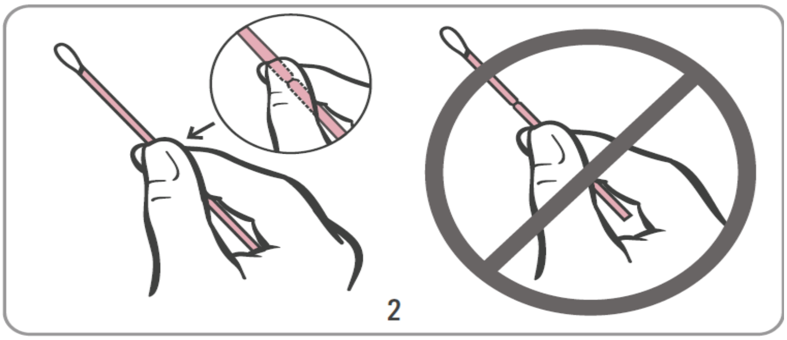

Hold swab as shown in Figure 2. Place thumb and index finger over the break line of swab.

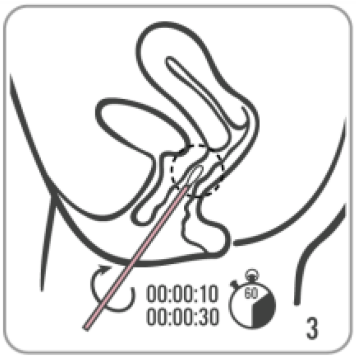

Spread the labia. Insert swab 3-5 cm into vagina. Or insert in rectum.
Turn for **5-10** seconds.

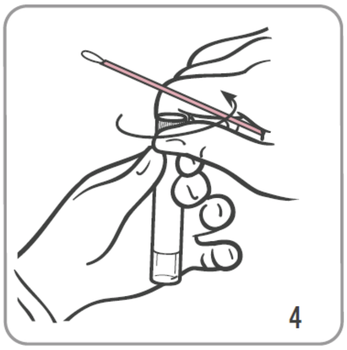

Unscrew the cap off the tube. With swab in hand.

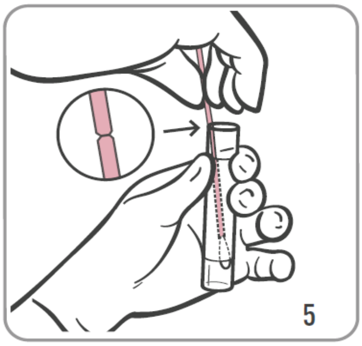

Insert the swab into the tube, into the liquid.

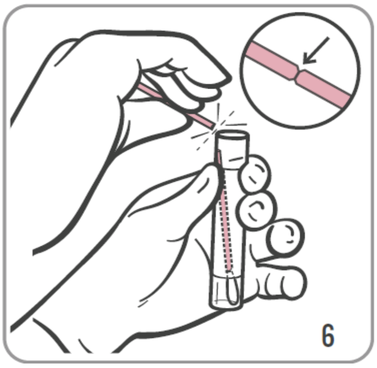

Break off at narrowing part.

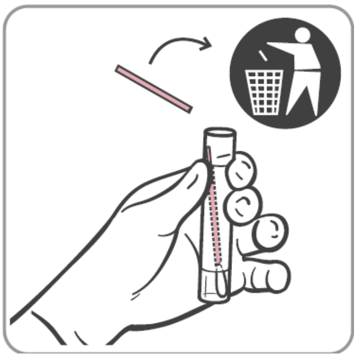

Discard remainer.

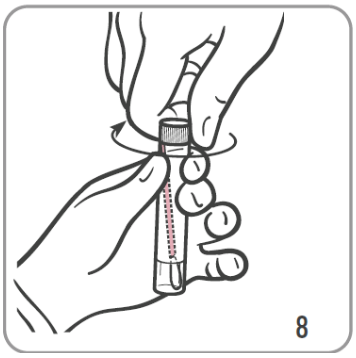

Screw cap firmly onto the tube.
