# Supplementary material for: Protocol of the PROMOTE study: characterization of the microbiome, the immune response, and one-carbon metabolism in preconceptional and pregnant women with and without obesity (an observational subcohort of the Rotterdam Periconception cohort)
Source: PLoS One. 2025 Apr 2;20(4):e0319618. doi: 10.1371/journal.pone.0319618 (PMC11964453; doi:10.1371/journal.pone.0319618)
Supplement: S3 File — (PDF) [file pone.0319618.s003.pdf]

S3 File. Manual for collecting stool sample PROMOTE study.

You have received a collection kit for taking a stool sample. In this stool sample, we will look at the composition of the gut microbiome. It is unknown whether a particular composition of the gut microbiome is beneficial or detrimental to maternal and child health in pregnancy.

Read the instructions completely before you start collecting stool and follow them carefully.
Preferably do the collection in the week before the 3D ultrasound in the 3rd trimester. If you have any questions, please contact promotestudie@erasmusmc.nl or tel. 06-81 17 76 05 (Monday till Friday 9am till 5pm).

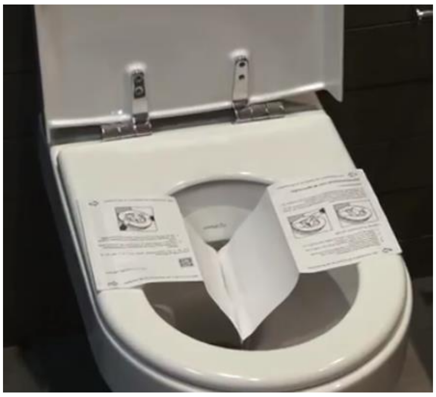

Figure 1 Fecescollector in toilet

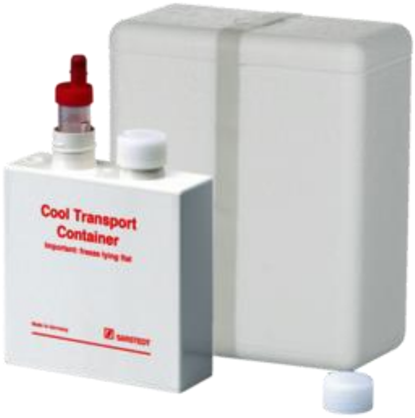

Figure 2 Cool Transport Container and Styrofoam box

Collection and storage

1. Collect feces with the feces collector (collection strip), see package for instructions (Do **not** drop feces into the water of the toilet) (Figure 1).
2. Take the tube and spatula to hand.
3. Open the tube and scoop a full spatula of feces into the tube 3-5 times with the spatula.
4. **Immediately** close the tube. Tighten the cap firmly to prevent the contents from leaking out.
5. The feces collector, with remaining feces, can then be flushed down the drain.
6. Remove the cool transport container from the Styrofoam (Figure 3). Unscrew the cap from the cooling holder, place the filled tube in here, then screw the cap back on. Place in the freezer **without** the Styrofoam tube. **Store** the Styrofoam tube for transport to the EMC!!!
7. Complete the collection form: name, collection date and time, medication use, events and the Bristol Stool Chart.

Transportation to Erasmus MC

1. Bring the cooling transporter with the filled tube of feces to the next study appointment
2. Leave the cooling holder in the freezer **as long as possible**: do not take it out until just before you leave home.
3. Place the pre-stored Styrofoam tube around the cooling transporter.
4. Take the completed form with you.
5. Hand the Styrofoam tube off to the examiner performing the ultrasound.
