# Supplementary material for: Protocol of the PROMOTE study: characterization of the microbiome, the immune response, and one-carbon metabolism in preconceptional and pregnant women with and without obesity (an observational subcohort of the Rotterdam Periconception cohort)
Source: PLoS One. 2025 Apr 2;20(4):e0319618. doi: 10.1371/journal.pone.0319618 (PMC11964453; doi:10.1371/journal.pone.0319618)
Supplement: S4 File — (PDF) [file pone.0319618.s004.pdf]

S4 File. Protocol PBMC isolation.

Everything should be prepared on <u>room temperature</u> unless otherwise specified in the steps.

1. 2 * 10 ml Heparin tubes

2. Dilute the blood 1:1 with PBS → this will give you approx. 40 ml of diluted blood

3. Add 4 ml of lymphoprep (RT) to 15 ml tubes (tubes needed: x ml diluted blood / 7.5)
   a. With 40 ml → 5 x 15 ml tubes.

4. Transfer 7.5 ml diluted blood (carefully) on Lymphoprep, equally distribute the remaining diluted blood over the tubes.
   a. Carefully place tubes in centrifuge, do NOT shake/mix!

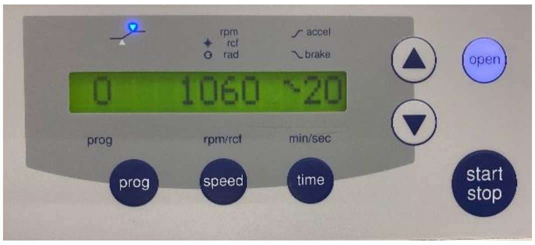

5. Centrifuge for 20 min at 1056 G at RT **<u>without brake (!)</u>**
   a. <u>Make sure you use rcf (*) = G!!!!!</u>
   b. <u>Brake = 0!!</u>

6. Discard the thrombocyte layer until ± 1 cm above the white PBMC ring → use suction machine

7. Using a pipet (5ml), carefully transfer the white PBMC ring to a new 15 ml tube
   Add maximally 2 rings per tube (of the same patient)

8. Fill the tubes with RPMI until a total volume of 10 ml, mix and centrifuge for 5 min at **<u>300 G (* rcf)</u>** with brake 9.

9. Discard the supernatant and <u>resuspend</u> the cell pellets from all tubes.
   a. Add 4 ml RPMI to the first tube, then transfer cell suspension to the next tube, repeat the same until all cells of a single patient are in a single tube.

b. Then take an amount of RPMI needed to get a total of 10 ml and rinse all tubes with this before adding it to the cell suspension; this will give you 10 ml of cell suspension (per patient).

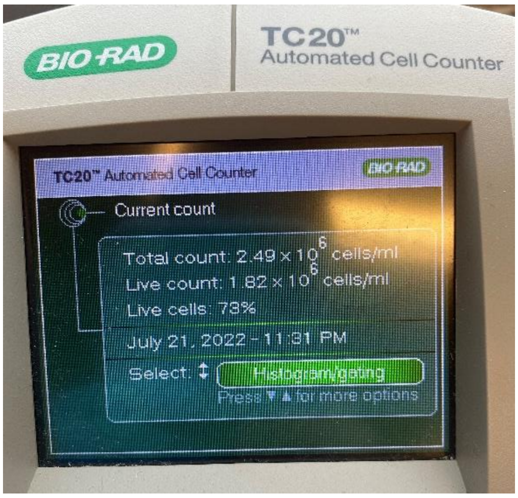

10. <u>Vortex the cell suspension</u>
    a. Mix 10µl Tryphan blue with 10µl cell suspension in appje
    b. Transfer the mixed suspension to a counting slice
    c. Count the cells with the BIO RAD Automated Cell Counter

11. Centrifuge the tube containing 10 ml of cell suspension at **<u>300 G (* rcf)</u>** 5 min with brake 9

12. Discard the supernatant and resuspend the cell pellet to a concentration of ± 20*10^6 PBMCs/ml using <u>cold</u> RPMI + 20% FCS. **Keep the cell suspension on ice (!)**

    …………………… cells * 1 ml / 20 * 10^6 = ……… ml

13. Dilute the cell suspension in RPMI + 20% FCS 1:1 with RPMI + 20% DMSO. Add the RPMI + 20% DMSO carefully. Do it dropwise: shake after adding a drop so that DMSO can enter the cells slowly DMSO reacts with RPMI and heat is produced, **so keep the cells on ice at all times (!)**

14. Transfer 1 ml of the cell suspension to each <u>cryovial</u> (10 * 10^6)
    End concentration = ± 10 * 10^6 cells in 10% FCS + 10% DMSO

15. Freeze the PBMCs in special freeze box (mr. Frosty).

    a. Note predictnumber + Moment of withdrawal on the cryovial.
        i. T0 = preconceptional
        ii. T1 = 1^e trimester
        iii. T2 = 2^e trimester
        iv. T3 = 3^e trimester
        v. T4 = Durante partu
        vi. T5 = Post partum

16. The next morning the PBMCs should be transferred to the liquid nitrogen freezer.
