# Supplementary material for: Protocol of the PROMOTE study: characterization of the microbiome, the immune response, and one-carbon metabolism in preconceptional and pregnant women with and without obesity (an observational subcohort of the Rotterdam Periconception cohort)
Source: PLoS One. 2025 Apr 2;20(4):e0319618. doi: 10.1371/journal.pone.0319618 (PMC11964453; doi:10.1371/journal.pone.0319618)
Supplement: S5 File — (PDF) [file pone.0319618.s005.pdf]

Category: Experimental Procedures
Author: Carien Bus
Version: 1

S5 File. Protocol PBMC isolation.
(Faecal) DNA extraction with RBB + Qiagen method

Labels: DNA extraction Sequencing

Materials
Gloves
1.5 ml eppendorf tubes (B74085-BIOplastics)
2.0 ml eppendorf tubes (623 201 Greiner)
2.0 ml screw cap tubes (B91211-BIOplastics)
screw caps (B91303-BIOplastics)
Glass beads 3mm
Silicium / Zirkonium beads 0.5 mm (11079101 BioSpec)
Nuclease free water (Promega-P1193)
RNase H (Promega- M428A)
Ethanol, >99% (Merck-)
Ammonium acetate (Merck-)
2-Propanol (Merck 1.01040)
Ethanol, pure (Merck 1.00983)
QIAamp DNA stool Minikit (Qiagen 51504)

Equipment
Thermoblock (<100oC)
waterbath (<100oC)
eppendorf centrifuge
eppendorf centrifuge with cooling (5417R)
Nanodrop-ND-1000
Beat Beater (Precellys 24, Bertan Technologies)
None specified

Solutions

Lysis buffer
500 mM NaCl,50 mM Tris-HCl (pH 8), 50 mM EDTA, 4 % SDS.
Make Stock solutions of the 4 components
o (1) 5 M NaCl
o (2) 1 M Tris-HCl (pH 8)
o (3) 0.5 M EDTA
o (4) 10% SDS
For 50 ml of lysis buffer you then need 5 ml of (1), 2.5 ml of (2), 5 ml of (3), 20 ml of (4) and 17.5 ml (filtered) water.

10 M ammonium acetate
Measure 192.7gr $C_2H_7NO_2$ and fill to 250ml with water.

70% ethanol
Take 35 ml pure ethanol and add 15 ml water

Experiment Settings
Step 1
Cell lysis:

1. Add 0,5g of 0,1mm zirconia beads and 4 glass beads (3 mm) to a 2,0 ml screw-cap tube. (Instead it is handier to put the beads in the "new 2 ml tube" described in step 5
instead, and do so the day before you start the whole procedure as part of your preparations)
2. Weigh 0,25 g of faeces into the tube, add the glass beads and add 1,0 ml of Lysis buffer. (If buffer is precipitated heat at +70°C or microwave very gently)
3. Treat sample in FastPrep at room temperature (RT) at 5,5 ms for 3 x 1min with 30 second pauses in between (or cool samples on ice in between).
4. Heat at 95°C for 15 min mix samples shaking by hand every 5 min.
5. Centrifuge at +4°C for 5 min at full speed (to pellet stool particles).
6. Transfer the supernatant into new 2ml eppendorf tube. Important, don't try to get all supernatant, take your loss.
7. Add 300 ul of fresh lysis buffer to the screw-cap lysis tube and repeat steps 3-5, then pool the supernatants.

Step 2
Precipitation of nucleic acids:

8. Add 260 ul of 10 M ammonium acetate to each lysate tube, mix well, and incubate on ice for 5 min
9. Centrifuge at 4°C for 10 min at full speed in a cooled centrifuge. Put the supernatant into a new 2ml eppendorf tube. Discard the pellet. Important: take your loss!
10. Again add 260 ul of 10 M ammonium acetate to each lysate tube, mix well, and incubate on ice for 5 min and centrifuge at 4°C for 10 min at full speed in a cooled centrifuge.
11. Transfer the supernatant to two 1,5 ml eppendorf tubes, add one volume of isopropanol and mix well, and incubate on ice for 30 min. Usually you must put about around
650 ul into each 1.5 ml eppendorf tube (and hence you must also add 650 ul isopropanol).
12. Centrifuge at RT for 15 min at full speed, remove the supernatant by decanting. Put the tubes upside down on a piece of paper for a moment to get rid of most of the
moisture (tapping a few times).
13. Wash nucleic acids pellet with 500 µl 70 % EtOH for 2 min. and dry the pellet to air with cups turned up-side down. After tapping no longer gets rid of any additional
moisture let them dry for about 5 more minutes. TAKE EXTREME CAUTION NOT TO LOSE THE PELLET DURING TAPPING (and if you do, find it and put it back).
14. Dissolve each nucleic acid pellet in 100 ul of AE buffer (from the kit), leave at 4°C overnight and pool the two aliquots.

Step 3
Removal of RNA, protein and purification (QIAmp DNA Mini Kit):

15. Add 2 ul of DNase-free RNase (10 mg/ml) and incubate at 37°C for 15 min.
a. Optional: In the meanwhile, put Buffer AW1 and AW2 on ice (step 18 and 19), to cool them down slightly.
16. Add 15 ul of proteinase K and 200 ul of Buffer AL mix well and incubate at 70°C for 10 min.
a. Do not mix proteinase K and Buffer AL in advance!
17. After incubation at 70 °C put the samples on ice for at least 1 minute (if you do not have a -80°C freezer for the alchol in the next step put the samples on ice longer).
18. Add 200 ul of icecold 100% EtOH (-80°C) and mix well. Transfer to a QIAmp column and centrifuge for 1 min at 14.000 rpm. (Exact speed is not that important, but it should be around 12000-14000).
19. Put the column in a new collection tube (Discard the flow through), add 500 l of Buffer AW1 and centrifuge for 1 min at RT at 14.000 rpm.

20. Put the column in a new collection tube (Discard the flow through), add 500 l of Buffer AW2 and centrifuge for 1 min at RT at14.000 rpm.
21. Put the column in a new collection tube (Discard the flow through), Dry the column by centrifugation at RT for 1 min, and dry the column after this by leaving the cup open for 1 min.
22. Add 100 ul of Buffer AE and incubate at room temperature for 1 min. Then centrifuge at 14.000 rpm for 1 min.
23. Re-use the elute with the DNA by decanting, incubate for 1 min. Then centrifuge at 14.000 rpm for 1 min. (take care not to lose any liquid, not all the elute needs to be put back into the column. If it is remaining as a droplet on the opening of the collection tube try to put it back into the collection tube).

Step 4
Check the purity of the DNA using a nano-drop

24. Check for the 260-280 and 260-230 values should preferentially be above 1.8. Also write down the amount of DNA. And check the curve.
25. Perform a PCR on the samples of which you have got doubts about the purity (low 260-280 and 260-230 values). Samples which are brown still will very likely not work at all (and will have very bad 260-280 and 260-230 values). Samples of which a PCR product is not obtained (check via gel electrophoresis) should get an additional purification step by using inhibitex and an aditional column assay.
26. Samples with a low DNA concentration (<10 ng/ul) will also have low 260-280 and 260-230 values, use a larger amount of sample in your PCR.
