# Supplementary material for: Protocol of the PROMOTE study: characterization of the microbiome, the immune response, and one-carbon metabolism in preconceptional and pregnant women with and without obesity (an observational subcohort of the Rotterdam Periconception cohort)
Source: PLoS One. 2025 Apr 2;20(4):e0319618. doi: 10.1371/journal.pone.0319618 (PMC11964453; doi:10.1371/journal.pone.0319618)
Supplement: S6 File — (PDF) [file pone.0319618.s006.pdf]

S6 File. Illumina capture sequencing.

DNase treatment:

- Centrifuge 110 µl sample for 10 minutes, 10.000 *g*
- Transfer 100 µl supernatant into new Eppendorf tube, take care not to include the pellet/debris
- Add 10 µl Turbo DNase (2U/µl; Ambion), 12 µl 10 X DNase buffer (Ambion) to the 100 µl supernatant
- Incubate 30 minutes at 37 ºC
- Add 200 lysis buffer + poly A (2500 ul binding buffer + 50 ul poly A)
- Add 50 ul prot K
- RNA extraction Roche High pure viral NA kit

*** A random cDNA synthesis was done with the RNA of samples:**

	One reaction
RNA	10
Random primer (10 µM)	1
dNTP's (10 mM)	1
Rnaspin plus (40 U/µl)	0.5

Program: BAS->

- 5 min. at 65°C
- On ice for 2 min or at 4°C in the PCR machine.

Added:

	One reaction
First strand buffer (5x) SSIV	4
DTT (100 mM)	1
Rnaspin plus (40 U/µl)	0.5
Superscript IV (200 U/µl)	0.5

Program: BAS->CDNA2

- **10 min. at 23°C**
- **10 min. at 50°C**
- **10 min. at 80°C**
- 2 min. on ice or at 4°C in the PCR machine

*** A Klenow reaction was done with the cDNA the samples:**

Added:

	Amount for 1 reaction (µl)
3'-5'Klenow DNA polymerase (5U/ul)	2

Program: BAS->DSDNA

- Incubated for 1 hour at 37°C
- Stopped the reaction by heating at 75°C for 10 minutes.

* **The sample Library was prepared:**

Note: Keep samples on ice before and after placement in a pre-cooled thermocycler.

	One reaction (µl)
100 ng DNA	20
water	15
KAPA Frag Buffer (10x) (Blue)	5
KAPA Frag Enzyme (Red)	10
Total	50

- Mix thoroughly
- Place in a pre-cooled thermocycler, set to instant incubate at 4°C. Then incubate the samples using the following program: BAS -> KAPfrag
  ∞4°C
  Step 1: 1 min. at 37°C
  Step 2: Hold at 4°C

- Transfer the sample to ice and proceed immediately to the next step

* **A End Repair and A-tailing was performed:**

- Prepare the End Repair and A-tailing mix

	One reaction (µl)
KAPA End Repair & A-Tailing Buffer	7
KAPA End Repair & A-tailing Enzyme Mix	3
Total	10

- Add 10 µl of End Repair and A-tailing Master Mix to each 50 µl fragmented sample
- Mix thoroughly
- Place on ice and immediately proceed to the next step
- Perform the End Repair and A-Tailing incubation in a thermocycler using the following program: BAS -> KAPENDRE

  Step 1: 30 minutes at 65°C
  Step 2: Hold at 4°C

- Proceed immediately to the next step

* **An Adapter Ligation was performed:**

- Prepare the Adapter Ligation Reaction Mix

	One reaction (µl)
PCR-grade water	5
KAPA Ligation Buffer	30
KAPA DNA Ligase	10
KAPA Dual-Indexed Adapter (15 µM) (1:100)	5
Total	50

- Add 50 µl of the Adapter Ligation Reaction Mix to 60 µl of the sample mix.
- Mix thoroughly
- Incubate the Ligation reaction at RT for 20 min.
- Place on ice and immediately proceed to the next step

Adapters used:

* **A Post-Ligation Cleanup was performed:**

- Perform the Post-Ligation Cleanup as follows
  (Ratio = 1:0.8, so for 1 µl sample : 0.8 µl beads)

	1ste wash One reaction (µl)	2nd wash One reaction (µl)
Ligation reaction	110	50
AMPure XP Beads	88	40
Total	198	90

- Mix thoroughly
- Incubate the samples at RT for 5 min. to allow the DNA to bind to the beads
- Place the samples in a magnetic particle collector to capture the beads. Incubate until the liquid is clear.
- Carefully remove and discard the supernatant
- Keep the sample on the magnet and add 200 µl of freshly-prepared 80% ethanol.
- Incubate at RT for ≥30 seconds
- Carefully remove and discard the supernatant
- Keep the sample on the magnet and add 200 µl of freshly-prepared 80% ethanol.
- Incubate at RT for ≥30 seconds
- Carefully remove and discard the supernatant. Try to remove everything without disturbing the beads
- Allow to dry at RT for ~4 min.
- Remove the tube from the magnet and resuspend the pellet in 53 µl elution buffer (10 mM Tris-HCL, pH 8.0) or PCR-grade water
- Incubate for 2 minutes at RT
- Pellet the beads on the magnet
- Transfer 50 µl supernatant to a fresh tube/well
- **Repeat, see table for amount of µl beads to use (40µl Ampure beads)**
- Proceed immediately to the next step.

* **<u>Double-Sided Size selection was performed:</u>**

	One reaction (µl)
Ligation reaction	50
AMPure XP Beads	30
Total	80

- Mix thoroughly
- Incubate the samples at RT for 5 min. to allow fragments larger than ~450 bp to bind to the beads
- Place the samples in a magnetic particle collector to capture the beads. Incubate until the liquid is clear.
- **Carefully transfer 80 µl of the supernatant containing library fragments smaller than ~600 bp to a new tube (fragments >600 bp bind to the beads and are not used).**
- Add 20 µl AMPure beads to the 80 µl of supernatant
- Thoroughly resuspend the beads by pipetting up and down
- Incubate the sample at RT for 5 min. to allow fragments larger than ~250 bp to bind to the beads
- Place the samples in a magnetic particle collector to capture the beads. Incubate until the liquid is clear.
- Carefully remove and discard the supernatant
- Keep the sample on the magnet and add 200 µl of freshly-prepared 80% ethanol.
- Incubate at RT for ≥30 seconds
- Carefully remove and discard the supernatant
- Keep the sample on the magnet and add 200 µl of freshly-prepared 80% ethanol.
- Incubate at RT for ≥30 seconds
- Carefully remove and discard the supernatant. Try to remove everything without disturbing the beads
- Allow to dry at RT for ≤1.5 min.
- Remove the tube from the magnet and resuspend the pellet in 23 µl elution buffer (10 mM Tris-HCL, pH 8.0) or PCR-grade water
- Incubate for 2 minutes at RT
- Pellet the beads on the magnet
- Transfer 20 µl supernatant to a fresh tube/well

* **<u>A Pre-Capture LM-PCR was performed:</u>**

- Prepare the following mix

	One reaction (µl)
KAPA HiFi HotStart readyMix (2X)	25
Library Amplification Primer mix	5
Total	30

- Add 30 µl pre-Capture LM-PCR master Mix to the samples and mix thoroughly
- Use the following program: BAS -> LM PCR

Step 1: 45 seconds at 98°C
Step 2: 15 seconds at 98°C
Step 3: 30 seconds at 60°C → 28x
Step 4: 30 seconds at 72°C
Step 6: 1 minute at 72°C
Step 7: Hold at 4°C
You can store the reaction at 4°C for up to 72 hours.

DAY2

*** The sample Library was purified:**

- Allow the AMPure XP Beads to warm up to room temperature for 30 minutes.
- Vortex the beads for 10 seconds.
- Add 40 µl beads to the 50 µl of sample library
- Vortex for 10 seconds
- Incubate the samples at RT for 5 min. to allow the DNA to bind to the beads
- Place the samples in a magnetic particle collector to capture the beads. Incubate until the liquid is clear.
- Carefully remove and discard the supernatant
- Keep the sample on the magnet and add 200 µl of freshly-prepared 80% ethanol.
- Incubate at RT for ≥30 seconds
- Carefully remove and discard the supernatant
- Keep the sample on the magnet and add 200 µl of freshly-prepared 80% ethanol.
- Incubate at RT for ≥30 seconds
- Carefully remove and discard the supernatant. Try to remove everything without disturbing the beads
- Allow to dry at RT for 5 minutes or until the beads are dry
- Remove the tube from the magnet and resuspend the pellet in 53 µl elution buffer (10 mM Tris-HCL, pH 8.0) or PCR-grade water
- Vortex for 10 seconds
- Incubate for 2 minutes at RT
- Pellet the beads on the magnet
- Transfer 50 µl supernatant to a fresh tube/well
- Repeat bead-purification

Purified amplified libraries can be stored at 4°C for 1-2 weeks or at -20°C

*** Concentration of the samples was measured with the Qubit:**

*** The quality of the samples was checked with the TapeStation:**

Protocol: See manufacturer

Check if there are adapters present in the samples. If so, preform another wash step.
If the concentration is to low, preform an extra LM-PCR with less cycli.

*** Make a pool of max 96 samples**

- Pool the samples with an end concentration of 1 µg DNA (1000 ng) (or a pool of for example 25nM) and measure the pool on Qubit and TapeStation.
